# Supplementary material for: Shikonin Derivatives Inhibit Inflammation Processes and Modulate MAPK Signaling in Human Healthy and Osteoarthritis Chondrocytes
Source: Int J Mol Sci. 2022 Mar 21;23(6):3396. doi: 10.3390/ijms23063396 (PMC8955849; doi:10.3390/ijms23063396)

Figure 3A

original

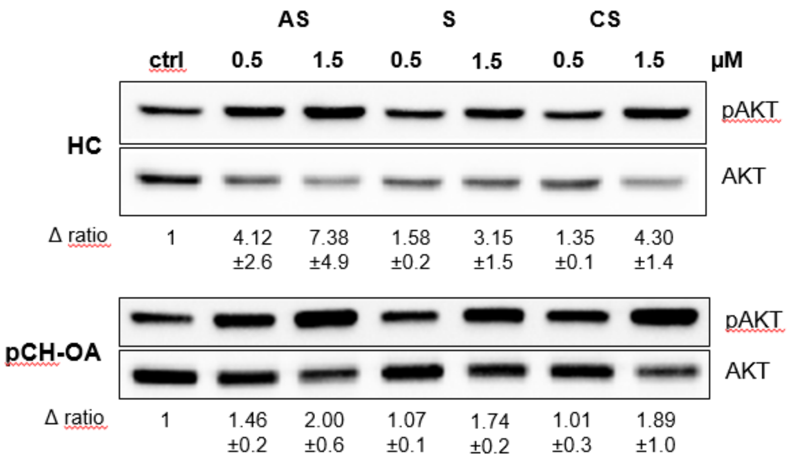

uncropped blots

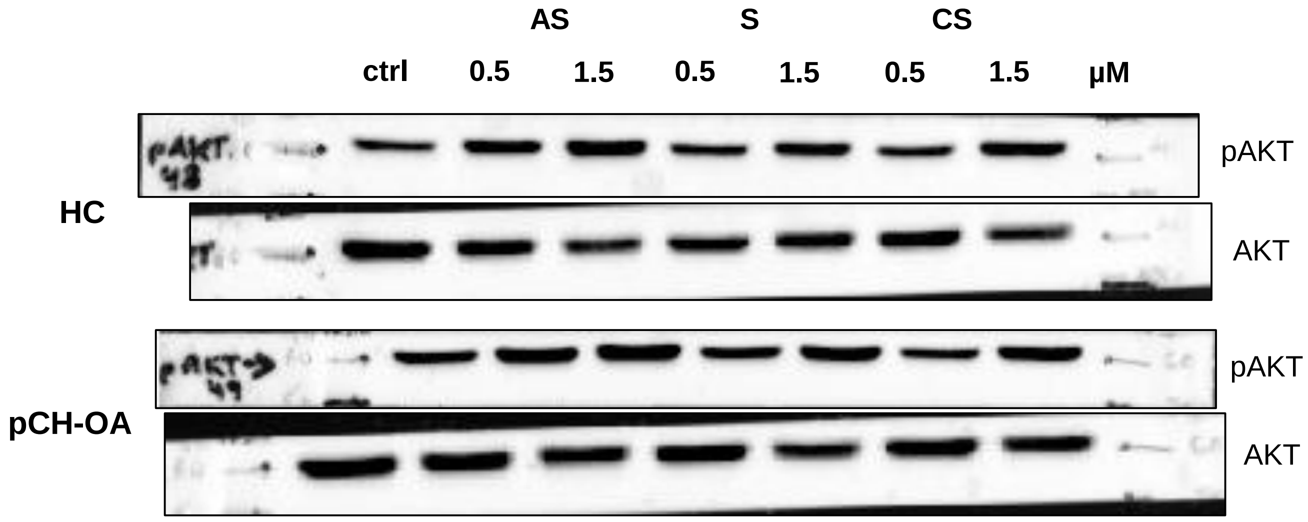

Figure 4A

original

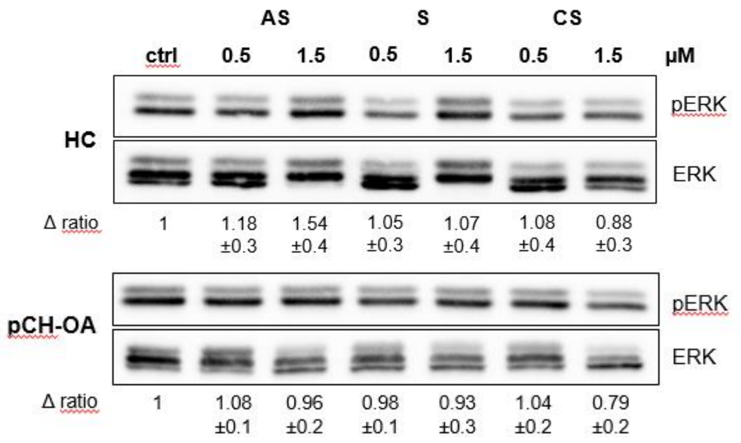

uncropped blots

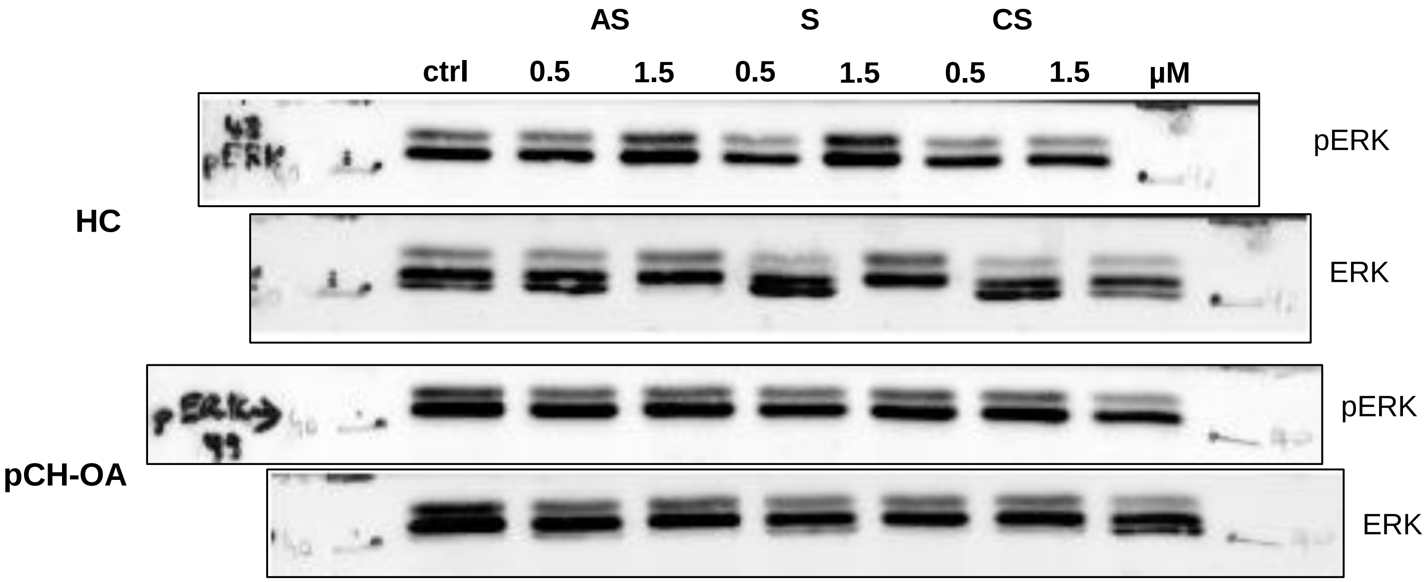

Figure 4A

original **uncropped blots**

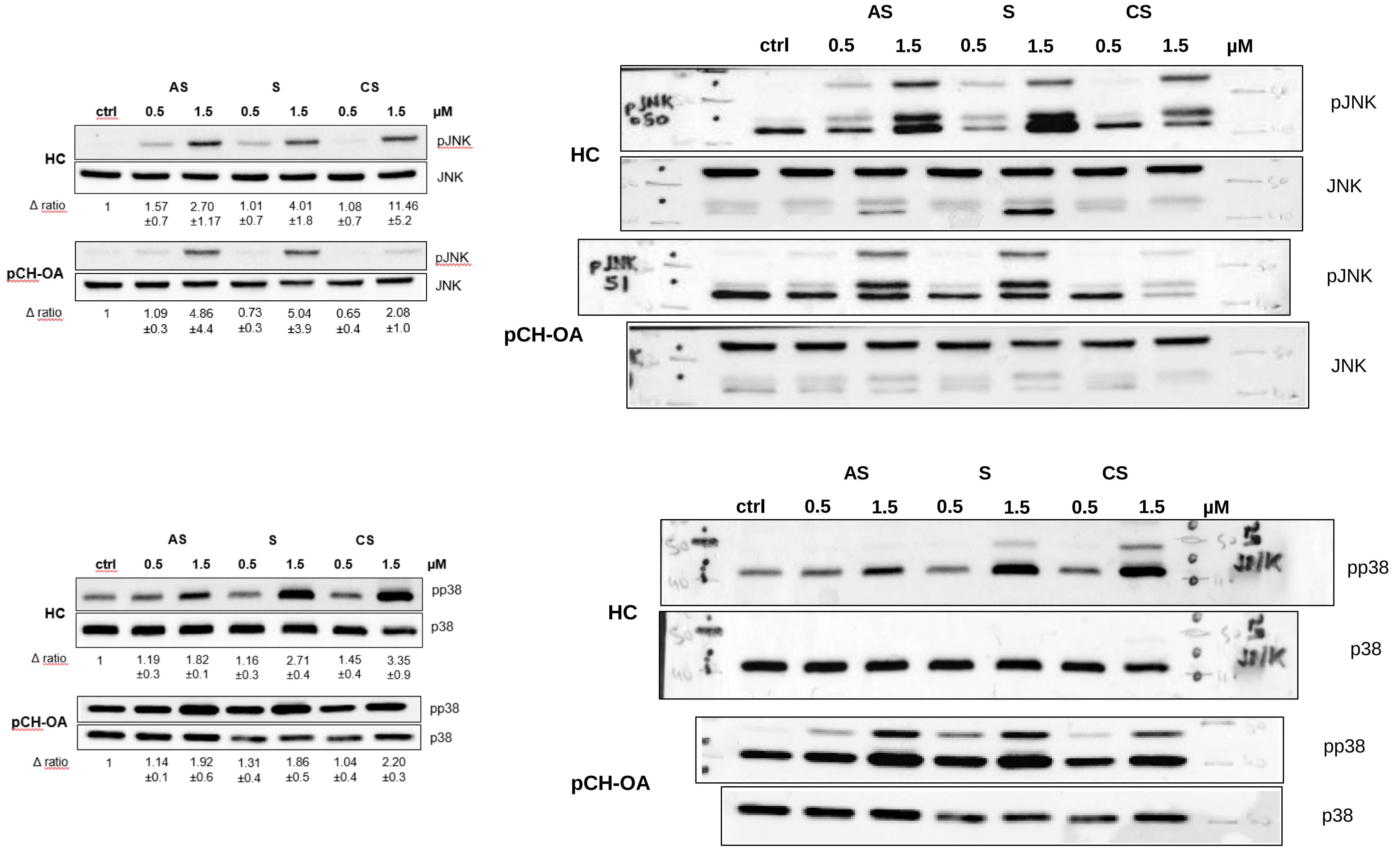

Figure 5A

original

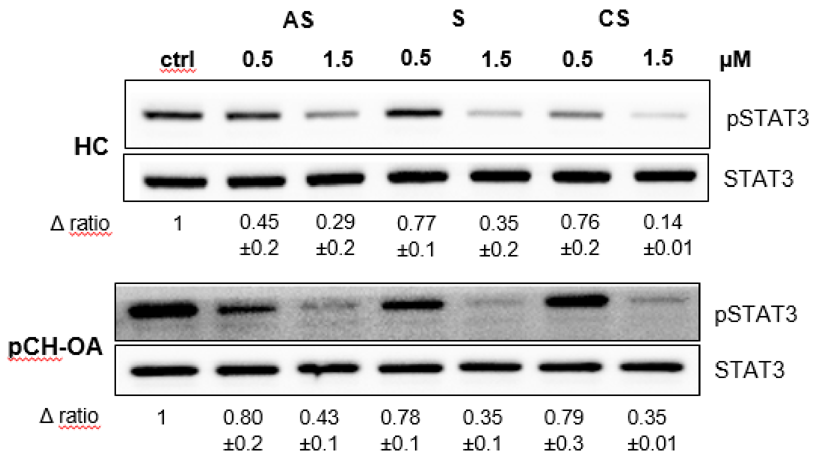

uncropped blots

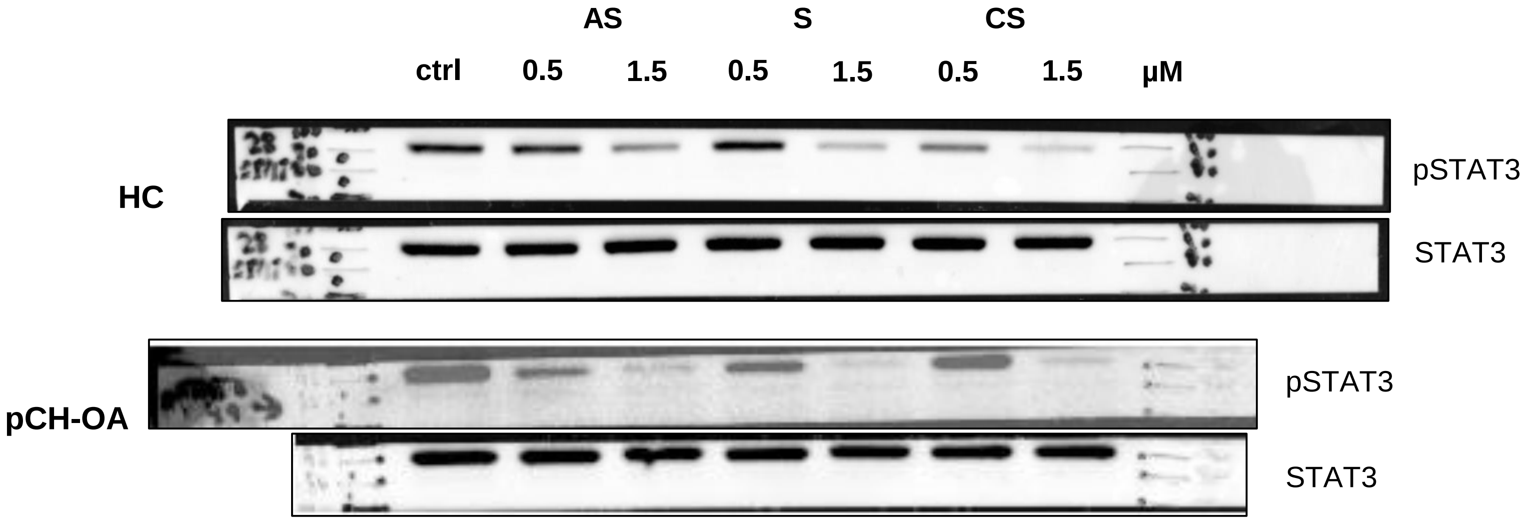

Figure 6A

original **uncropped blots**

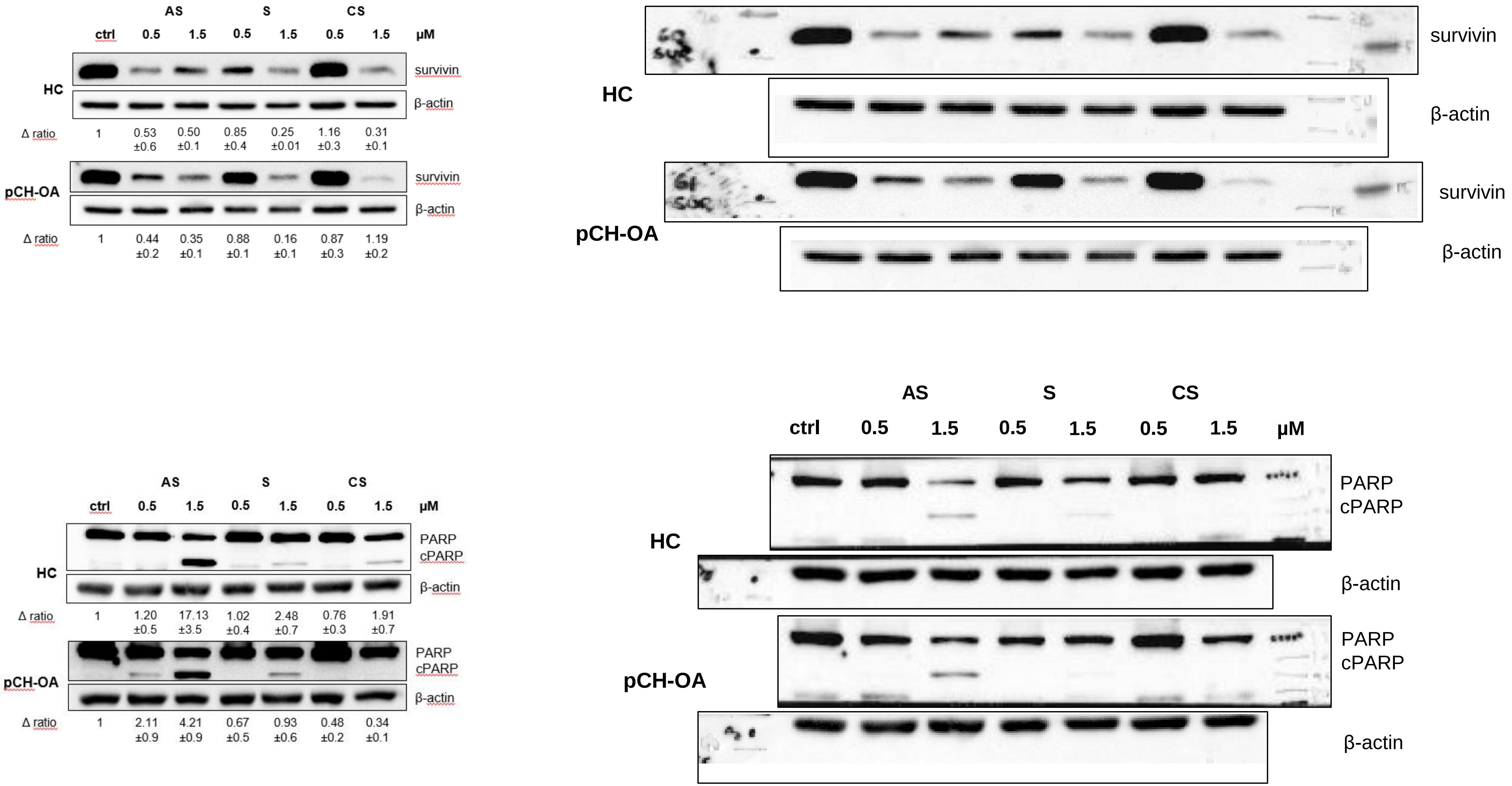

Figure 6A

original

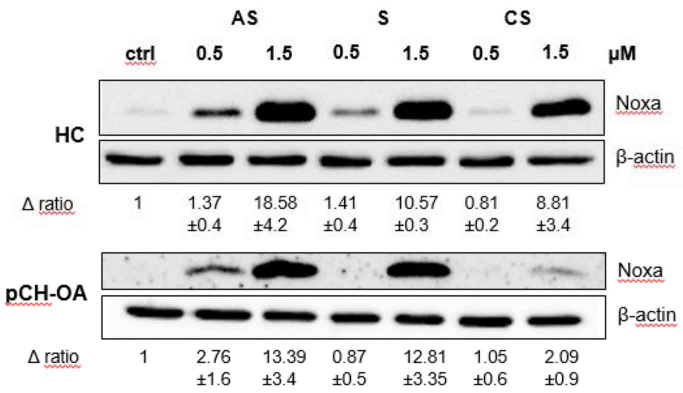

uncropped blots

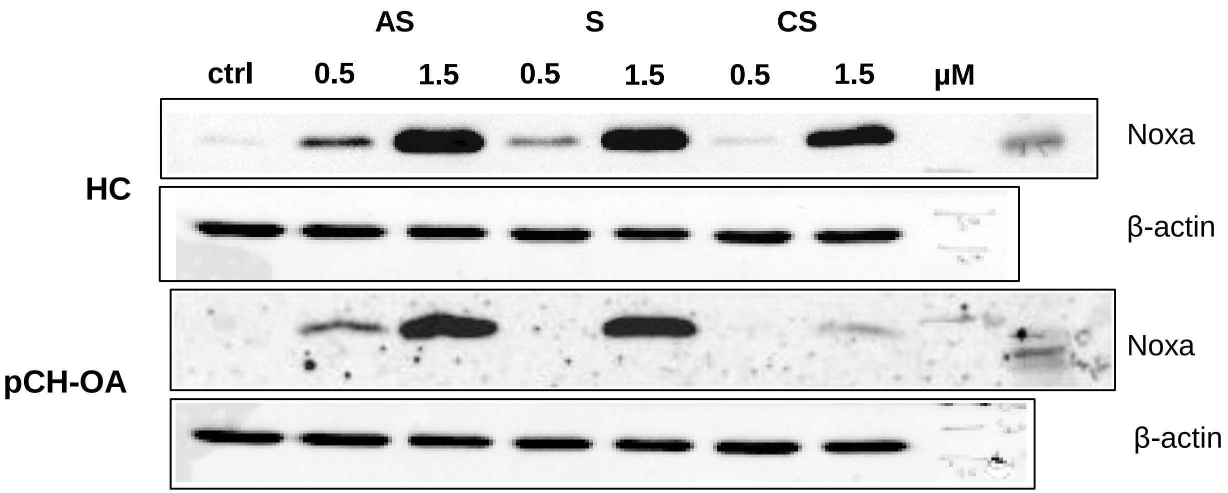

Supplement: Supplementary file 1 [file ijms-23-03396-s001.zip › ijms-1607961-supplementary.pdf]
